# Role of CXCR4 as a Prognostic Biomarker Associated With the Tumor Immune Microenvironment in Gastric Cancer

**DOI:** 10.3389/fcell.2021.654504

**Published:** 2021-09-08

**Authors:** Yuyang Gu, Wenyue Gu, Rongrong Xie, Zhi Chen, Tongpeng Xu, Zhenghua Fei

**Affiliations:** ^1^Department of Oncology, The First Affiliated Hospital of Wenzhou Medical University, Wenzhou, China; ^2^Yancheng Third People’s Hospital, The Sixth Affiliated Hospital of Nantong University, Yancheng, China; ^3^Department of Oncology, The First Affiliated Hospital of Nanjing Medical University, Nanjing, China

**Keywords:** CXCR4, tumor microenvironment, gastric cancer, ESTIMATE algorithm, CIBERSORT algorithm

## Abstract

**Background:** Gastric cancer (GC) is a leading cause of cancer-related deaths worldwide, accounting for high rates of morbidity and mortality in the population. The tumor microenvironment (TME), which plays a crucial role in GC progression, may serve as an optimal prognostic predictor of GC. In this study, we identified CXC motif chemokine receptor 4 (*CXCR4*) as a TME-related gene among thousands of differentially expressed genes (DEGs). We showed that *CXCR4* can be used to predict the effect of immunotherapy in patients with GC.

**Methods:** GC samples obtained from The Cancer Genome Atlas (TCGA) were analyzed for the presence of stroma (stromal score), the infiltration of immune cells (immune score) in tumor tissues, and the tumor purity (estimate score) using the ESTIMATE (Estimation of STromal and Immune cells in MAlignant Tumor tissues using Expression data) algorithm. DEGs were sorted based on differences in the values of the three scores. Furthermore, Gene Ontology (GO) and Kyoto Encyclopedia of Genes and Genomes (KEGG) analyses were performed to determine the biological processes and pathways enriched in these DEGs. The correlations of scores with clinicopathological features and overall survival (OS) of patients with GC were assessed by the Kaplan–Meier survival and Cox regression analyses. Through subsequent protein–protein interaction (PPI) network and univariate Cox regression analyses, CXCR4 was identified as a TME-related gene. Gene Set Enrichment Analysis (GSEA) was performed to assess the role of CXCR4 in the TME of GC. The CIBERSORT algorithm was used to further explore the correlation between tumor-infiltrating immune cells (TIICs) and CXCR4. Finally, the TISIDB database was used to predict the efficacy of immunotherapy in patients with GC.

**Results:** We extracted 1231 TME-related DEGs and by an overlapping screening of PPI network and univariate Cox regression, CXCR4 was identified as a biomarker of TME, which deeply engaged in immune-related biological processes of gastric cancer and have close association with several immunocompetent cells.

**Conclusion:** CXCR4 may be a useful biomarker of prognosis and an indicator of the TME in GC.

## Introduction

Gastric cancer (GC) is one of the most common malignant tumors of the alimentary system, with growing incidences worldwide. Globally, GC is the sixth most frequently diagnosed cancer and the third leading cause of cancer-related deaths (Sitarz et al., 2018). More than 1,000,000 new cases are diagnosed, and approximately 783,000 deaths occur annually (Bray et al., 2018; Global Burden of Disease Cancer Collaboration, Fitzmaurice et al., 2019). Although surgical resection remains the primary curative treatment for GC, the incidence of postoperative tumor recurrence is high. In particular, the 5-year survival rate of patients with stage II, III, and IV GC is approximately 31, 13, and 3%, respectively (Akhondi-Meybodi et al., 2017). Despite remarkable progress in treatment modalities in recent years, the mortality rate of patients with GC remains high (Rawla and Barsouk, 2019). Therefore, it is necessary and urgent to develop novel strategies for the early diagnosis and prognostic prediction to reduce the high mortality and recurrence rates of patients with GC.

Previous investigations have demonstrated that the characteristics of the tumor microenvironment (TME) are closely associated with the progression and prognosis of GC (Wang et al., 2019). The significance of the TME in cancer initiation and progression has drawn increasing attention in recent years. Research has shown that the TME is an active promoter of cancer progression, as opposed to its previous designation as a silent bystander during cancer (Bussard et al., 2016). Emerging evidence has indicated that the TME, which is mainly composed of the extracellular matrix, stromal cells, blood vessels, and lymphatic networks, plays a key role in tumor development and metastasis (Hanahan and Coussens, 2012; Junttila and de Sauvage, 2013; Quail and Joyce, 2013). The type and proportion of stromal cells are related to the physiological state of the TME (Alkasalias et al., 2018). Moreover, tumor-infiltrating immune cells (TIICs), such as CD8+ T cells, regulatory T cells (Tregs), and tumor-associated macrophages (TAMs), positively affect the clinical outcome of patients with various malignancies, including melanoma, lung cancer, breast cancer, and GC (Adams et al., 2013; Massi et al., 2015; Bremnes et al., 2016; Jiang et al., 2017). The dynamic interplay between stromal cells and immune cells in the TME involves several cellular events and physiological processes (Lee et al., 2014). Further investigations of various components and pathways of GC in the TME may facilitate targeted therapy.

Recently, ESTIMATE (Estimation of STromal and Immune cells in MAlignant Tumor tissues using Expression data), a novel algorithm, has been developed to calculate stromal and immune scores, which are used to assess the extent of stromal and immune cells infiltrating into tumor tissues. The ESTIMATE algorithm helps present a better picture of the numbers of stromal and immune cells in the TME (Yoshihara et al., 2013). Thus, based on the scores calculated by ESTIMATE, the clinical outcomes of patients with GC may be predicted (Liu et al., 2018).

In this article, we collected the gene expression profiles of patients with GC from The Cancer Genome Atlas (TCGA) and used the ESTIMATE algorithm to calculate immune and stromal scores of the TME in GC. Moreover, we investigated the correlation between the risk scores obtained from differentially expressed genes (DEGs) and the clinicopathological characteristics of patients with GC. Furthermore, we constructed a protein–protein interaction (PPI) network and conducted a functional enrichment analysis of the identified DEGs to explore their potential correlations with TIICs.

## Materials and Methods

### Microarray Data Collection and Processing

From the TCGA dataset, transcriptome and relevant clinical data of 373 patients with GC (343 tumor samples vs. 30 normal samples) were collected, and the ESTIMATE algorithm was employed to evaluate the composition of the TME. The results were represented as three scores, namely immune score, stromal score, and estimate score, corresponding to the proportion of immune cells, stromal cells, and both immune and stromal cells, respectively. All genes of tumor samples were ranked by their expression levels, and DEGs were screened out using the “limma” package in R. DEGs were identified based on the following criteria: an absolute value of log2 fold change (| log2FC|) > 1 and false discovery rate (FDR) < 0.05. Furthermore, the “VennDiagram” package was used to screen for genes with similar expression levels in both stromal and immune cells. The “pheatmap” package was used to produce heatmaps of TME-related DEGs.

### Functional Enrichment Analysis of TME-Related DEGs

tumor microenvironment-related DEGs were performed Kyoto Encyclopedia of Genes and Genomes (KEGG) and Gene Ontology (GO) analysis, which revealed the function of DEGs in the biologic process, molecular function, and showing the pathway enrichment result. The “ggplot2,” “enrichplot,” and “clusterProfiler” packages in R were used to perform GO and KEGG analyses. Statistical significance was set at *P* < 0.05 and *q* < 0.05.

### Correlation Between Scores With Clinicopathological Characteristics and Survival

The clinicopathological characteristics of each sample were evaluated by the Wilcoxon rank-sum and Kruskal–Wallis rank-sum test, clarifying the correlation between scores and the clinical stage. Samples were divided into high- and low-score groups by compared to media value and executed survival analysis. R packages “survival” and “survminer” were applied and *P* < 0.05 was identified as significant difference.

### PPI Network and Cox Regression Analysis

Next, to explore the relationship among DEGs, the STRING platform^1^ was used to establish a PPI network, and nodes were employed to reconstruct the network with the confidence of interactive relationship greater than 0.95. The Cytoscape software was used to identify the top 30 hub genes. Univariate Cox regression was performed using the “survival” package in R to select DEGs associated with the prognosis of GC. The top 50 genes ranked according to log-rank test *P*-values in univariate Cox analysis are shown in the plot. Finally, based on the results of the intersection analysis of the PPI network and Cox regression analysis, only the CXC motif chemokine receptor 4 (*CXCR4*) gene was found to meet all the above-mentioned metrics.

### Correlation Between CXCR4 Expression and Clinicopathological Characteristics

Kaplan–Meier survival analysis was performed to illustrate the differences in the overall survival (OS) between the GC groups with low and high expression. Next, correlation analysis was performed between clinical characteristics and *CXCR4* expression levels, which were contrasted by univariate analysis. Statistical analysis was performed using SPSS 22.0, and statistical significance was set at *P* < 0.05.

### Further Analysis of the Relationship Between CXCR4 and Tumor Immunoreaction

To explore the role of CXCR4 in the TME of GC, Gene Set Enrichment Analysis (GSEA) was performed to verify the results of KEGG pathway enrichment analysis using GSEA version 3.0 (Broad Institute, Cambridge, MA, United States). Differences were considered significant if NOM *P*-value < 0.05 and FDR < 0.25. In addition, to determine the relative abundance of TIICs in GC samples, the extent of infiltration was estimated using the CIBERSORT algorithm. Samples with *P* < 0.05 were identified to have significantly different immune cell infiltration between the two groups. Furthermore, correlation analyses between the expression of *CXCR4* and immune cell infiltration in the TME were performed. Additionally, the correlation of the expression levels of CXCR4 with those of immune checkpoint molecules in GC was identified by the TISIDB web portal^2^. Statistical significance was set at *P* < 0.05.

## Results

### Identification and Functional Analysis of DEGs

**FIGURE 1:**
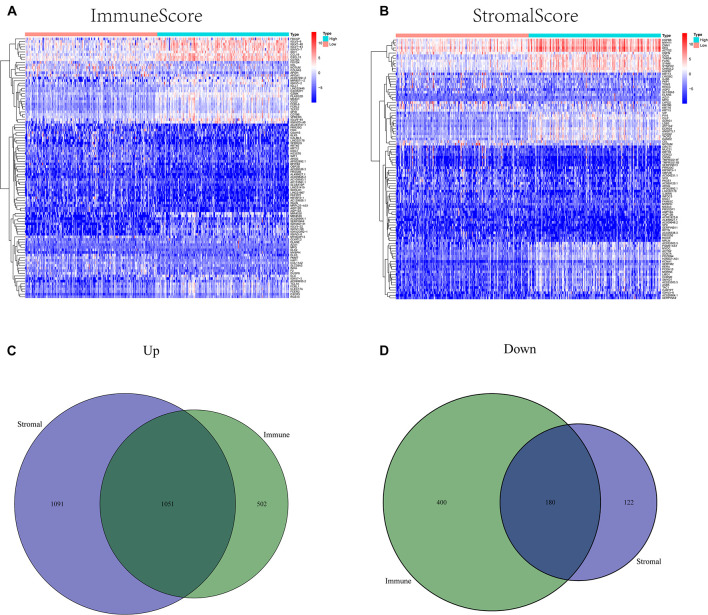
Heatmaps and Venn plots of differentially expressed genes (DEGs). **(A)** A heatmap of immune-related DEGs between the high- and low *CXCR4* expression groups. **(B)** A heatmap of stromal-related DEGs between the high- and low *CXCR4* expression groups. **(C)** A Venn diagram of commonly upregulated DEGs in the stromal and immune components. **(D)** A Venn diagram of commonly downregulated DEGs in the stromal and immune components.

**FIGURE 2:**
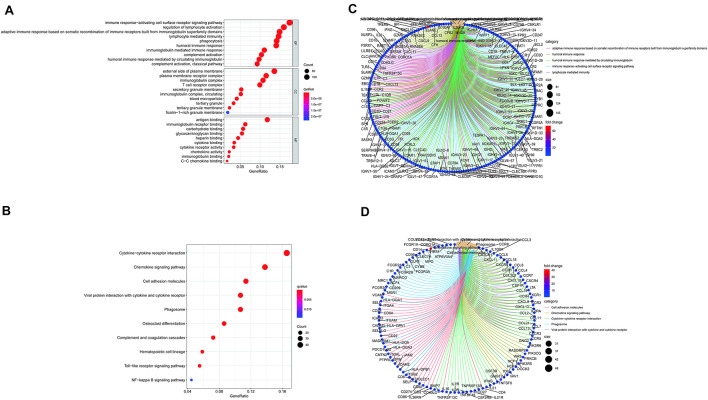
Gene Ontology (GO) and Kyoto Encyclopedia of Genes and Genomes (KEGG) enrichment analysis of differentially expressed genes (DEGs) **(A–D)**.

ImmuneScore, StromalScore, and ESTIMATEScore were dissected by Kaplan–Meier survival analysis. The high- and low-score samples were analyzed and compared to determine the differences in gene expression patterns in immune and stromal components. A total of 2143 DEGs were obtained based on the immune score, out of which 1553 genes were upregulated and 580 genes were downregulated (Figures 1A,C,D). Similarly, 2454 DEGs were acquired based on the stromal score, out of which 2152 genes were upregulated and 302 genes were downregulated (Figures 1B,C,D). Furthermore, the Venn plot identified 1051 upregulated genes and 180 downregulated genes in both the immune and stromal components. These 1231 DEGs were identified as TME-related DEGs. The results of GO analysis demonstrated that these DEGs were mostly engaged in immune-related functions, such as the regulation of lymphocyte activation and lymphocyte-mediated immunity (Figures 2A,C). Moreover, the results of KEGG analysis demonstrated the involvement of DEGs in certain immune-related functions, including cytokine-cytokine receptor interaction and chemokine signaling pathway (Figures 2B,D).

### Correlation Between Scores and Clinicopathological Features of Patients With GC

**FIGURE 3:**
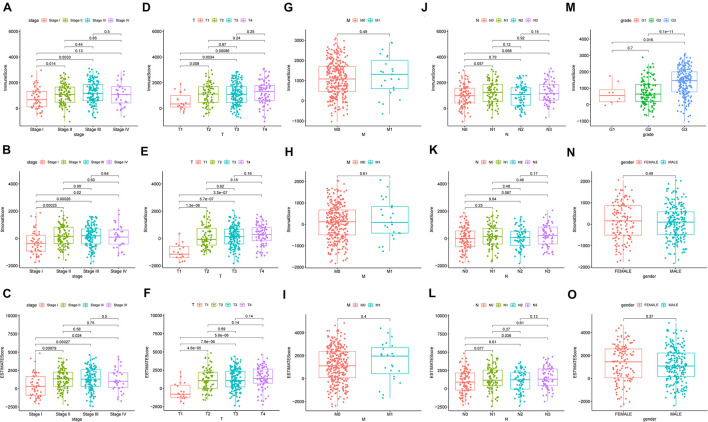
Correlation of clinicopathological characteristics with immune, stromal, and estimate scores **(A–O)**.

**FIGURE 4:**
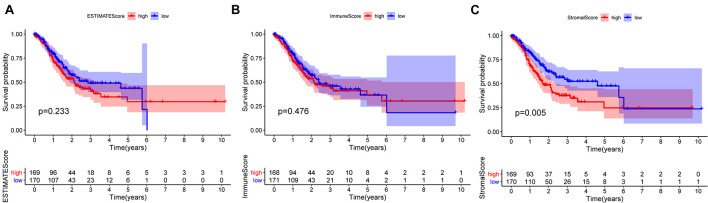
Correlation of each estimate, immune, and stromal score with the survival outcome of patients with gastric cancer **(A–C)**.

To ascertain the correlation between stromal and immune scores with the clinicopathological characteristics of patients with GC, commensurable clinical features of patients with GC acquired from TCGA were analyzed. The immune score was found to be significantly and positively correlated with the T stage of the tumor (*P* = 0.00086) (Figure 3D) and the cancer grade (*P* = 0.016) (Figure 3M). Moreover, the stromal scores were significantly correlated with the T stage of the tumor (*P* < 0.001) (Figure 3E) and the cancer stage (*P* = 0.02) (Figure 3B). Furthermore, the estimate score was closely associated with the T and N stages of the tumor (*P* = 0.024, *P* < 0.001, and *P* = 0.036, respectively) (Figures 3C,F,L). However, all three scores were not significantly associated with the M stage of the tumor (*P* = 0.49, *P* = 0.61, and *P* = 0.04, respectively) (Figures 3G–I) while immune score was not statistically significantly correlated with N stage (*P* = 0.068) (Figure 3J) or the stage of tumor (*P* = 0.13) (Figure 3A). Neither stromal score or estimate score were associated with gender (*P* = 0.49, *P* = 0.37, respectively) (Figures 3N,O). Besides, there was no significant correlation between stromal score and N stage (*P* = 0.067) (Figure 3K). The correlation of immune, stromal, and estimate scores with patient survival was analyzed by the Kaplan–Meier survival method, and the correlation of each score with the survival rate was assessed. As shown in Figure 4C, the amount of the stromal constituent was negatively correlated with the OS of patients with GC (*P* = 0.005). However, the immune and estimate scores had no significant correlation with the OS (Figure 4A, *P* = 0.233; Figure 4B, P = 0.476). These results suggest that the stromal components in the TME are significantly associated with the prognosis of patients with GC.

### Intersection Analysis of Univariate Cox Regression and PPI Network

**FIGURE 5:**
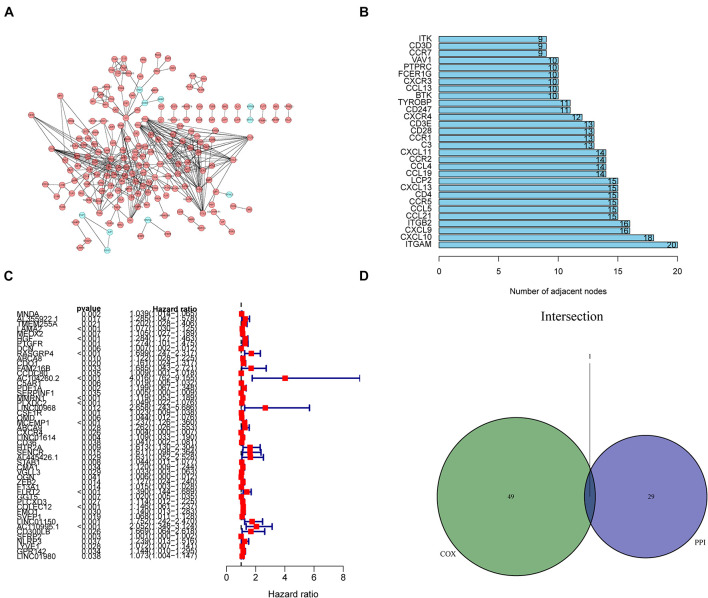
Protein–protein interaction (PPI) network and univariate Cox regression analysis. **(A)** Construction of the PPI network of 1231 differentially expressed genes (DEGs). **(B)** The top 30 genes ranked by the number of adjacent nodes of the PPI network. **(C)** Results of univariate Cox regression analysis with selected DEGs, with the top 50 genes displayed in the forest plot. **(D)** A Venn diagram showing *CXCR4* as the most commonly expressed DEG in gastric cancer by combination analysis of the top 30 genes in the PPI network and the top 50 prognostic genes from the results of Cox regression analysis.

Next, we thoroughly investigated the interactions among DEGs. We constructed a PPI network using the STRING database in Cytoscape. The correlation between each DEG and the top 30 genes ranked by the nodes was displayed in Figures 5A,B. The results of univariate Cox regression analysis showed that 50 genes were associated with the prognosis of GC (Figure 5C). The results of intersection analysis between the 30 hub genes and 50 prognostic DEGs revealed *CXCR4* as the only overlapping gene (Figure 5D).

### Association Between CXCR4 Expression With Clinicopathological Factors and Disease Progression

**FIGURE 6:**
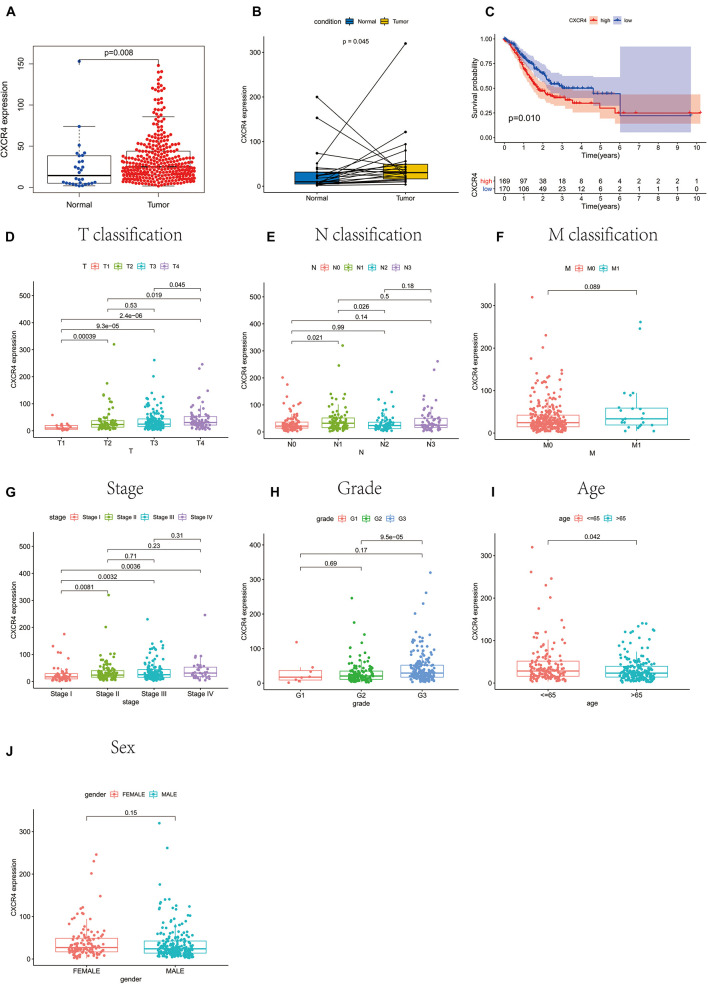
The relationship between *CXCR4* expression with survival rate, clinical and pathological characteristics of patients with gastric cancer. **(A)** unpaired samples, **(B)** paired samples, **(C)** survival rate, **(D)** T classification, **(E)** N classification, **(F)** M classification, **(G)** stage, **(H)** grade, **(I)** age, and **(J)** sex.

In the present study, GC samples were divided into high CXCR4 and low CXCR4 groups using the median expression value of *CXCR4* as the threshold value. Survival analysis showed that GC patients with low *CXCR4* expression had a longer survival time than those with high *CXCR4* expression (Figure 6C, *P* = 0.010). Results of the Wilcoxon rank-sum test demonstrated that the level of CXCR4 in tumor tissues was significantly higher than that in healthy tissues in both paired or unpaired samples (Figure 6B, *P* = 0.045; Figure 6A, *P* = 0.008). In addition, the expression of CXCR4 was significantly different between different age groups (Figure 6I, *P* = 0.042). Additionally, the level of CXCR4 was inextricably linked to the T stage (Figure 6D, *P* = 2.4e-06) and the stage of cancer (Figures 6G, *P* = 0.0036). However, there were no significant differences between the expression level of CXCR4 and the M stage (Figures 6F, *P* = 0.089), N stage (Figures 6E, *P* = 0.089), grade (Figure 6H, *P* = 0.17), or sex (Figures 6J, *P* = 0.15).

### Role of CXCR4 in the TME of Gastric Cancer

**FIGURE 7:**
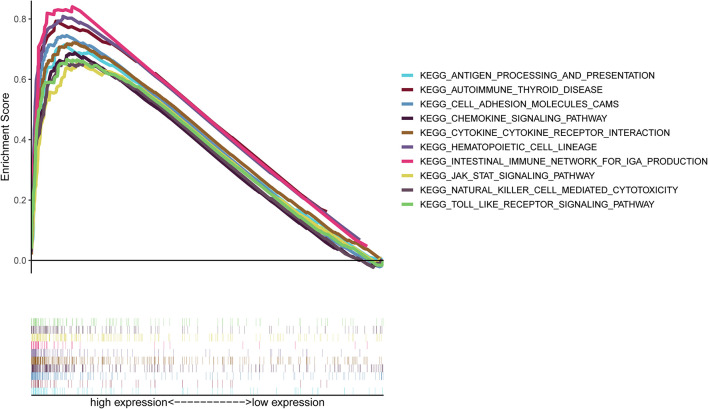
Gene set enrichment analysis of *CXCR4*.

**FIGURE 8:**
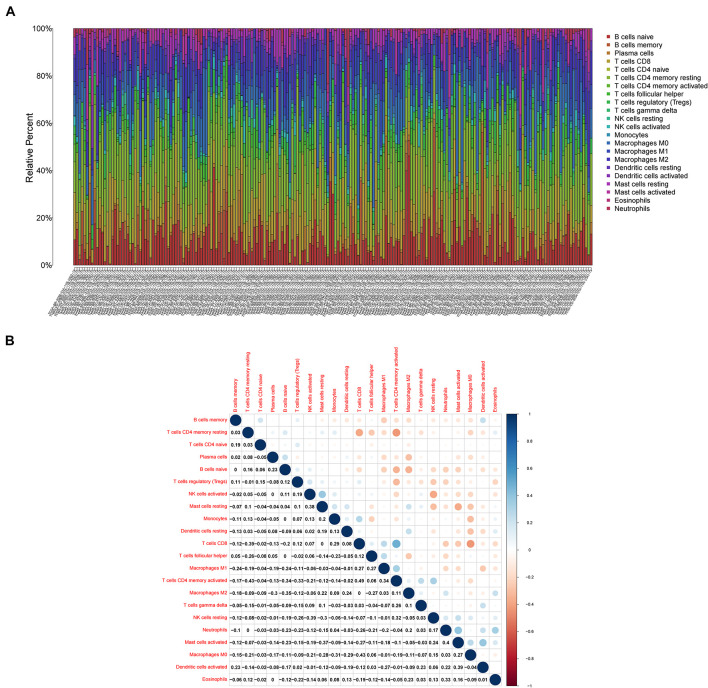
Profiles of tumor-infiltrating immune cells (TIICs) in gastric cancer (GC) samples and their correlation analysis. **(A)** Barplot showing the proportion of 21 different types of TIICs in GC tumor samples. **(B)** A heatmap showing the correlation between 21 different types of TIICs.

**FIGURE 9:**
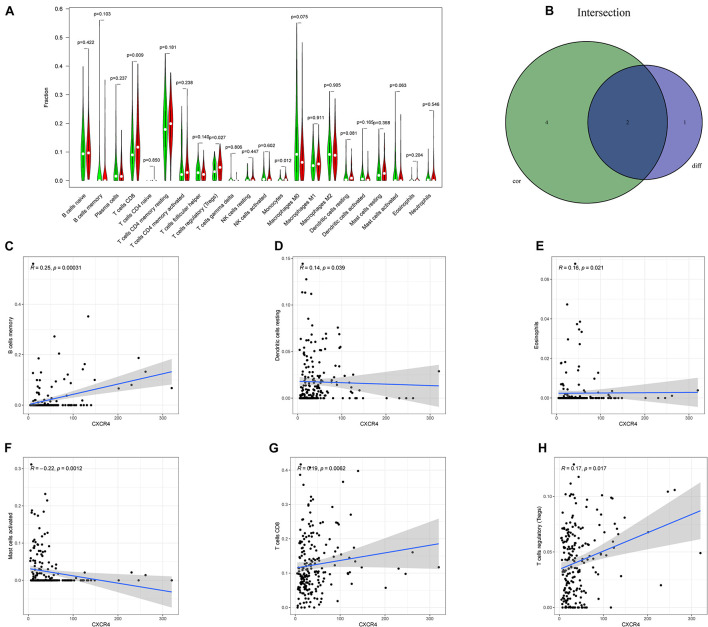
Relation between the expression of *CXCR4* and proportion of tumor-infiltrating immune cells (TIICs). **(A)** A violin plot showing the differences in the proportions of 21 different types of immune cells in GC tumor samples with low or high *CXCR4* expression. **(B)** A Venn plot displaying the differences and correlation between two types of TIICs associated with *CXCR4* expression. **(C–H)** A scatter plot showing the correlation of the proportions of TIICs with *CXCR4* expression.

**FIGURE 10:**
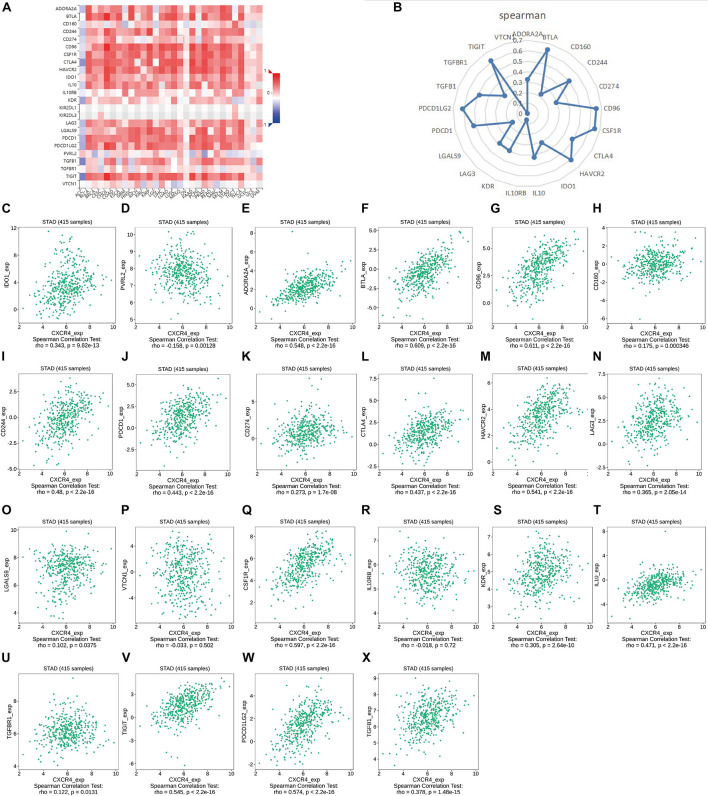
Correlation analysis of the expression of *CXCR4* expression with that of immune checkpoint genes. **(A,B)** Correlation analysis of the expression levels of *CXCR4* with those of 22 common immune checkpoint genes in gastric cancer. **(C–X)** The relationship between the expression of *CXCR4* with that of *IDO1*, *PVRL2*, *ADORA2A*, *BTLA*, *CD96*, *CD160*, *CD244*, *PDCD1*, *CD274*, *CTLA4*, *HAVCR2*, *LAG3*, *LGALS9*, *VTCN1*, *CSF1R*, *IL10RB*, *KDR*, *IL10*, *TGFBR1*, *TIGIT*, *PDCD1LG2*, and *TGFB1*.

As shown in Figure 7, *CXCR4* was mainly engaged in immune-related activities, such as an intestinal immune network for IgA production, JAK-STAT signaling pathway, natural killer cell-mediated cytotoxicity, and Toll-like receptor signaling pathway. Using the CIBERSORT algorithm, we identified the infiltrating profiles of 22 different types of immune cells in tumor tissues (Figures 8A,B). A total of six types of TIICs were found to be strongly correlated with *CXCR4* expression in the TME of GC cells (Figure 9). Immune cells such as memory B cells, resting dendritic cells, CD8+ T cells, monocytes, and Tregs were positively related with the expression of *CXCR4*, while decreased activation of mast cells was negatively correlated with *CXCR4* expression. Furthermore, we characterized the interactions between *CXCR4* with 22 immune control genes. As shown in Figure 10, the expression level of *CXCR4* was positively correlated with that of 20 immune checkpoint molecules, including *CD274*, *CTLA-4*, and *LAG3*, among others. Therefore, these results indicate that CXCR4 plays an important role in the immune evasion of GC cells.

## Discussion

Great advances in whole-genome sequencing have facilitated the development of molecular classification systems and treatment strategies for cancer. In the present study, we identified *CXCR4* as a TME-related gene associated with survival and TMN-stage classification in GC samples gathered from TCGA database.

CXC motif chemokine receptor 4, the receptor for chemokine CXCL12/SDF-1 and a member of the G protein-coupled receptor superfamily, is overexpressed in various types of solid cancers, including non-small-cell lung cancer (NSCLC), breast cancer, colorectal cancer, and GC. Previous studies have demonstrated that CXCR4 accelerates the metastasis, invasion, growth, and therapeutic resistance of cancer (Zhao et al., 2010; Otsuka et al., 2011; Ying et al., 2012; Mukherjee and Zhao, 2013; Xu et al., 2018). Moreover, CXCR4 affects the migration of GC cells via the ERK/Akt signaling pathway (Cheng et al., 2017). Cheng et al. (2020) found that the positive crosstalk between CXCR4 and EGFR promotes GC metastasis via the NF-kB pathway. Another study revealed that CXCR4 activates the NF-kB pathway and upregulates the expression of serine proteinase inhibitor clade B member 3, thereby facilitating the migration and invasion of GC cells (Gong et al., 2020). CXCR4 also plays a critical role in tumor angiogenesis in GC by activating the JAK2/STAT3 (Zhang et al., 2017). Furthermore, it promotes the proliferation and invasion processes via the Wnt/β-catenin pathway (Lin et al., 2017). Thus, inhibition of CXCR4 can disrupt multiple processes that facilitate the growth and spread of GC tumors. Therefore, given its multiple functions, CXCR4 may prove to be a promising target for immunotherapy.

Tumor progression is determined not only by cancer cells but also by the TME, which is the internal environment of malignant tumor progression. The TME can reduce the resistance of cancer cells to chemotherapy and immunotherapy (Russi et al., 2019). Epithelial-mesenchymal transition (EMT), a major modulator of tumor metastasis, may be involved in the interaction between tumor cells and the TME (Zheng et al., 2015; Suarez-Carmona et al., 2017; Hsieh et al., 2018). Compelling evidence indicates that CXCR4 regulates tumor EMT together with the c-MET signaling pathway (Quail and Joyce, 2013). The stromal component, another key component of the TME, primarily consists of cancer-associated fibroblasts (CAFs), which drive the growth, metastasis, and malignancy of cancer cells (Cheng et al., 2018). One study showed that CXCL12 secreted from CAFs promotes GC cell invasion by enhancing the clustering of integrin β1 in GC cells (Daisuke et al., 2015). In addition, CXCL12 mainly stems from the stromal part, which directly stimulates the proliferation and migration of CXCR4-expressing cells. Thus, the specific interaction between stromal cells and tumor cells might be one of the causes of drug resistance. Fortunately, AMD300, a CXCR4 inhibitor approved by the United States Food and Drug Administration (FDA), has already been shown to disrupt tumor-stromal interactions, sensitizing cancer cells to docetaxel-based chemotherapy in prostate cancer (Domanska et al., 2012). Currently, AMD300 is the most frequently used drug targeting the CXCL12-CXCR4 axis in clinical trials for solid gastrointestinal tumors, and therefore, CXCR4 might be a promising candidate target for GC immunotherapy.

In this study, the CIBERSORT algorithm was used to analyze the proportion of TIICs. The results showed that Tregs, dendritic cells, eosinophils, and CD8+ T cells were significantly positively correlated with *CXCR4* expression in patients with GC, which is concurrent with our hypothesis that *CXCR4* may be the hub gene of the TME in GC. Previous studies have revealed that CD8+ T cells are associated with poor prognosis in GC (Thompson et al., 2017). Similarly, Tregs are known to play an immunosuppressive role in the TME. Further reports have indicated that Tregs derived from patients with cancer usually express diverse chemokine receptors, which contributes to their migration into tumors in response to the signals stemming from the TME (Yan et al., 2011). However, some studies argued that Treg infiltration predicts favorable outcomes for patients with GC. For example, Li et al. (2016) reported that lower FOXP3+ and GARP+ Treg levels after neoadjuvant chemotherapy are associated with good outcomes in progressive GC. Thus, Treg infiltration may play a subtle yet vital role in GC progression. Nevertheless, more subset and related molecule regulation mechanisms of Tregs should to investigated thoroughly to better evaluate the prognosis of patients with GC (Liu et al., 2019).

Moreover, we have attempted to characterized the correlation between the expression levels of *CXCR4* with that of immune checkpoint molecules. The results revealed moderate positive correlations between the expression of *CXCR4* with that of PD-L1 (CD274) or CTLA4 in GC, which, in some ways, may be exploited for improving the efficacy of immunotherapy. Previous evidence indicated that suppression of CXCR4 promotes anti-PD-1/PD-L1 efficacy by reshaping the TME in hepatocellular carcinoma (Chen et al., 2015). A similar conclusion was observed in osteosarcoma, where Feig et al. (2013) showed that the SDF-1/CXCR4 axis facilitates the accumulation of myeloid-derived suppressor cells in the TME to abate the response to anti-PD-1 therapy. Another study discovered that in patients with colorectal cancer liver metastases, the expression of *CXCR4 CXCR7*, *TLR2/TLR4*, and *PD-1/PD-L1* was significantly increased in metastatic liver tissues compared to unaffected liver tissues (D’Alterio et al., 2016). Interestingly, our study found that CXCR4 was closely related to the Toll-like receptor signaling pathway (Figure 10). However, more experiments should be conducted to elaborate the interaction of CXCR4, TLRs, and PD-1/PD-L1 in GC. Furthermore, Pep R, a novel CXCR4 antagonist, has been found to enhance the efficiency of anti-PD-1 in various models (D’Alterio et al., 2019). X4-136, another CXCR4 inhibitor, could serve as a monotherapy or combined with immune checkpoint inhibitors in the treatment of melanoma and renal cell carcinoma (Saxena et al., 2020). In addition, a few studies have demonstrated that the combined blockade of CXCL12-CXCR4 and PD-1- PD-L1 pathways could provide survival benefits by regulating the TME of various solid tumors (Feig et al., 2013; Wu et al., 2019; Zeng et al., 2019; Seo et al., 2019) shedding light on CXCR4/PD-1-targeting combination therapy in GC. Given these advances, although the correlation between the expression of CXCR4 and PD-1/PD-L1 was moderate, we theorized that it has far-reaching clinical implications and relevance, which needs further experimental verification.

This study has some limitations that need to be acknowledged. First, as the clinical data were mainly acquired from TCGA database, result biases were unavoidable. Additionally, we did not conduct experimental research to inspect the function of CXCR4 in GC. The combined application of CXCR4 blocker and PD-1 inhibitor may prolong the survival time of GC. However, more evidence is needed to prove the mechanism of combined immunotherapy.

In summary, we employed the ESTIMATE algorithm to identify genes that were associated with the TME in GC samples gathered from TCGA database. Consequently, CXCR4 was discovered as a promising prognostic target for patients with GC. Nevertheless, more experimental research is warranted to explore the underlying molecular mechanisms and potential clinical value of CXCR4 for the early diagnosis of tumor micrometastasis.

## Conclusion

Overall, the ESTIMATE algorithm was used to calculate the immune, stromal, and estimate scores of GC samples acquired from TCGA. Stromal and immune cells that infiltrated into the TME were closely related to tumor growth. We identified a few TME-related DEGs, out of which *CXCR4*, was significantly associated with the regulation of the immune-active status in the TME. Therefore, CXCR4 might be a latent biomarker in GC, which determines the efficacy of cancer immunotherapy. The results of the present study may provide new insights into the development of effective therapeutic strategies targeted against GC.

## Data Availability Statement

Publicly available datasets were analyzed in this study. This data can be found here: the datasets analyzed for this study can be found in the TCGA (https://cancergenome.nih.gov/) and ImmPort (https://www.immport.org).

## Author Contributions

TX and ZF conceived and devised the study and gave aid in writing the manuscript. YG and WG performed the data analyses and contributed to the writing of the manuscript. ZC and RX reviewed the original manuscript. All authors reviewed the manuscript.

## Conflict of Interest

The authors declare that the research was conducted in the absence of any commercial or financial relationships that could be construed as a potential conflict of interest.

## Publisher’s Note

All claims expressed in this article are solely those of the authors and do not necessarily represent those of their affiliated organizations, or those of the publisher, the editors and the reviewers. Any product that may be evaluated in this article, or claim that may be made by its manufacturer, is not guaranteed or endorsed by the publisher.

## Footnotes

1
http://string-db.org/

2
http://cis.hku.hk/TISIDB/index.php
